# Changes in the Treatment of Some Physico-Chemical Properties of Cassava Mill Effluents Using *Saccharomyces cerevisiae*

**DOI:** 10.3390/toxics5040028

**Published:** 2017-10-16

**Authors:** Sylvester Chibueze Izah, Sunday Etim Bassey, Elijah Ige Ohimain

**Affiliations:** Department of Biological Sciences, Faculty of Science, Niger Delta University, Wilberforce Island, P.M.B. 071, Yenagoa, Bayelsa State, Nigeria; sonebass@yahoo.com (S.E.B.); eohimain@yahoo.com (E.I.O.)

**Keywords:** cassava mill effluents, environmental contaminants, *Saccharomyces cerevisiae*, treatment

## Abstract

Cassava is majorly processed into gari by smallholders in Southern Nigeria. During processing, large volume of effluents are produced in the pressing stage of cassava tuber processing. The cassava mill effluents are discharged into the soil directly and it drain into nearby pits, surface water, and canals without treatment. Cassava mill effluents is known to alter the receiving soil and water characteristics and affects the biota in such environments, such as fishes (water), domestic animals, and vegetation (soil). This study investigated the potential of *Saccharomyces cerevisiae* to be used for the treatment of some physicochemical properties of cassava mill effluents. *S. cerevisiae* was isolated from palm wine and identified based on conventional microbiological techniques, viz. morphological, cultural, and physiological/biochemical characteristics. The *S. cerevisiae* was inoculated into sterile cassava mill effluents and incubated for 15 days. Triplicate samples were withdrawn from the setup after the fifth day of treatment. Portable equipment was used to analyze the in-situ parameters, viz. total dissolved solids (TDS), pH, dissolved oxygen (DO), conductivity, salinity, and turbidity. Anions (nitrate, sulphate, and phosphate) and chemical oxygen demand (COD) were analyzed using spectrophotometric and open reflux methods respectively. Results showed a decline of 37.62%, 22.96%, 29.63%, 20.49%, 21.44%, 1.70%, 53.48%, 68.00%, 100%, and 74.48% in pH, conductivity, DO, TDS, salinity, sulphate, nitrate, phosphate, and COD levels respectively, and elevation of 17.17% by turbidity. The study showed that *S. cerevisiae* could be used for the treatment of cassava mill effluents prior to being discharged into the environment so as to reduce the pollution or contamination and toxicity levels.

## 1. Introduction

Cassava is one of the major staple foods consumed globally. Presently, Nigeria is the largest producer of cassava in the world [1,2], accounting for about 54 million metric tonnes, as of the end of the 2014 economic year [3]. Cassava processing is a major agricultural business in Nigeria. The cultivation and processing of cassava into several products is a major source of livelihood to several families, especially in the Southern region. Cassava is processed into several products such as high-quality cassava flour, fufu, and lafun. Furthermore, cassava processing in Nigeria is predominantly carried out by smallholders who utilize rudimentary equipment for processing.

Cassava processing generates three major waste streams: air (gaseous emissions), liquid (cassava mill effluents), and solid (cassava peels, seivates) [1]. Specifically, large volume of wastewater are generated from the dewatering/pressing zone during cassava processing. This waste water is typically discharged into the environment (soil and water) without treatment in developing countries like Nigeria.

Cassava mill effluents have been reported to contain high total dissolved solids (799 mg/L), total suspended solids (789 mg/L) [4], total solids (5600 mg/L), total hardness (75.00 mg/L) [5], conductivity (1550 µS/cm) [6], acidic pH (2.5–5.07) [3,4,5,6,7], low dissolved oxygen (1.10–2.60 mg/L), and high chemical oxygen demand (320–365 mg/L) [7]. Cassava mill effluents also contain ions including anions and cations (i.e., alkaline earth metals and heavy metals). Some of the physicochemical constituents of cassava mill effluents discharged into the environment often exceed the permissible level specified by FEPA [8] for all categories of industrial effluents to be discharged into the environment.

Cassava mill effluents alter the receiving environmental quality such as soil and surface water. Some of the notable parameters altered include microbial, physical, and chemical, including heavy metal characteristics [9,10,11,12,13,14,15,16,17,18,19,20,21,22,23,24]. Cassava mill effluents also have an offensive and unpleasant odor that could disturb people residing close to cassava mills. In addition, cassava mill effluents on the receiving water affect the downstream utilization of such water. Authors have also reported that cassava mill effluents induce mortality, behavioral response, and changes in enzymatic, haematological, and histopathological parameters in fisheries, especially *Clarias gariepinus* [25,26]. According to Ero and Okponmwense [27], the toxicity of nitrogenous compounds such as nitrite—nitrogen, nitrate—nitrogen could be derived from cassava tend to affect fishes in the aquatic ecosystem. Instances of cassava mill effluents toxicity on domestic animals and livestock such as goat and sheep have been reported by Ehiagbonare et al. [18], and on vegetation by Otunne and Kinako [28].

Due to the impact of cassava mill effluents on the environment and biodiversity (fisheries, livestock, microorganism, and vegetation), there is the need to treat the effluents prior to discharge. Several other industrial effluents are discharged into the environment with little or no treatment in Nigeria [29], especially from non-oil and gas activities.

Several technologies (chemical, physical, and biological) are available for the treatment of effluents, depending on the source. Some of the factors that determine the choice of treatment technology include constituents and characteristics of the effluents. Microbes typically aid in the reduction in most constituents of an effluent through the mineralization of the available nutrients. Okoduwa et al. [29] reported that the use of yeast such as *S. cerevisiae* and *Torulaspora delbrueckii* could rapidly reduce inorganic compounds found in tannery waste water.

The treatment of cassava mill effluents prior to discharge could lower the impacts on the receiving environment. Therefore, the aim of this study is to isolate *S. cerevisiae* from palm wine and use it for the treatment of cassava mill effluents.

## 2. Materials and Methods

### 2.1. Sample Collection

Triplicate raw cassava mill effluents containing palm oil were collected from a smallholder cassava processor employing the manual process at Ndemili in Ndokwa West Local Government Area of Delta state, Nigeria. Samples were collected using 4 L clean containers and were transported to the laboratory using ice packs. The samples were used immediately upon arrival in the laboratory.

### 2.2. Isolation of Saccharomyces cerevisae Used for the Study

Palm wine was purchased from a palm wine vendor in Rumuomasi, Port Harcourt, Rivers state, Nigeria. Serial dilution of the palm wine was carried out based on the method previously described by Benson [30] and Pepper and Gerba [31]. About 1 mL of the palm wine samples was pipetted into 9 mL of sterile distilled water and shaken properly. The dilution was made up to 10^−6^. Then, 1 mL of the diluents was inoculated into the sterilized petri dish. The prepared sterilized potato dextrose agar supplemented with chloramphenicol was poured onto the petri dish. The petri dish was rotated on the bench several times (clockwise and anticlockwise) to achieve even spreading. The agar plate was allowed to solidify and was incubated inverted at room temperature 30 °C ± 4 °C for 3–5 days. The resultant isolate was streaked in a fresh potato dextrose agar plate supplemented with chloramphenicol. The resultant isolates were preserved in an agar slant prior to further analysis.

### 2.3. Identification of Saccharomyces cerevisae Used for the Study

The yeasts were identified using conventional microbiological techniques based on their cultural, morphological, and physiological/biochemical characteristics, as described by Kurtzman and Fell [32], APHA [33], Benson [30], and applied by Iwuagwu and Ugwuanyi [34], Abioye et al. [35,36], and Okoduwa et al. [29]. The resultant characteristics was compared with the guide provided by Ellis et al. [37].

#### 2.3.1. Identification Using Lacto-Phenol Cotton Blue Stain Methylene Blue

The methylene blue analysis was carried out based on the method described by Benson [30]. Wet mount preparation of the isolate was made on a clean, grease-free glass slide using methylene blue indicator. A sterile wire loop was used to collect a loop of the isolate, which was placed in methylene blue indicator and properly mixed. Then, immersion oil was added and the slide was covered with cover slip. The slide was viewed under oil immersion. Similarly, a wet mount preparation was also made using lactose-phenol cotton blue stain and viewed under the microscope.

#### 2.3.2. Carbon Fermentation Test

The carbon fermentation method was specifically carried out following the method previously applied by Iwuagwu and Ugwuanyi [34]. The carbon fermentation basal medium was comprised of 4.5 g of powdered yeast extract (LAB M, Heywood, United Kingdom), 7.5 g of peptone (LAB M, Heywood, United Kingdom), and 26.7 mg of bromothymol blue indicator, which was prepared with 2% sugar (maltose, glucose, sucrose, lactose) in 1 L of distilled water and then sterilized. An amount of 6 mL of the prepared medium was dispensed into the fermentation tubes under aseptic condition. Durham tubes were introduced into the tubes to trap gases and were tightly capped. After inoculating the isolates into peptone water and incubating for 48–72 h, 0.2 mL of inoculum was aseptically dispensed into the tubes and incubated at room temperature for 2 weeks. During the incubation period, the medium was shaken twice in 24 h. Color-change from green to yellow-like and gas production indicates a positive result. The results were compared with the scheme of Kurtzman and Fell [32], Ellis [37].

#### 2.3.3. Carbon Assimilation Test

The carbon assimilation test was carried out based on the methodology described by Kurtzman and Fell [32] and as applied by Iwuagwu and Ugwuanyi [34]. Yeast Nitrogen Base agar slants were supplemented with 2% sugar (maltose, glucose, sucrose, lactose), then the slant medium was inoculated with 0.2 mL of the isolate incubated in peptone water for 48–72 h. The slants were incubated at room temperature and inspected every 3 days for 21 days. Heavy growth after some days of incubation is an indication of strong carbon assimilation.

#### 2.3.4. Growth Based on Temperature Using Glucose–Peptone–Yeast Extract Broth

The growth based on temperature was carried out based on the method previously applied by Iwuagwu and Ugwuanyi [34], with slight modifications. The test was carried out using a glucose–peptone–yeast extract broth prepared with 20 g of glucose, 10 g of peptone, and 5 g of yeast extract, and dissolved in 1000 mL of distilled water. Then, 6 mL of the medium was dispensed into the test tubes and capped with cotton wool. The medium was sterilized by autoclaving and allowed to cool. Furthermore, 0.2 mL of the isolate inoculated into peptone water and incubated for 48–72 h was aseptically introduced into the sterilized medium and incubated at room temperature. The tubes were inspected daily for growth.

### 2.4. Preparation of S. cerevisiae Inoculum and Cassava Mill Effluent for Yeast Growth

The isolate was inoculated into peptone water and incubated for 3–4 days. Then, the sample was inoculated into a slant for preservation. The incubated inoculum was used for the cassava mill effluent treatments. The cassava mill effluents were filtered using double layered-muslin cloth, then were boiled and allowed to cool.

### 2.5. Effluents Treatment Studies

The yeast growth was carried out based on the method previously described by Abioye et al. [35] and Okoduwa et al. [29], with slight modifications. A 100 mL quantity of the prepared cassava mill effluents was measured into a 250 mL Erlenmeyer’s flask under aseptic conditions, and 10 mL of *S. cerevisiae* inoculum was added into the flask. The flask was capped with cotton wool wrapped with aluminum foil paper. A control was set up without the *S. cerevisiae* inoculum. A total of fifteen (comprising of 12 treatments and 3 controls) 250 mL Erlenmeyer’s flasks were used for the study. Growth was determined for 15 days at 5-day intervals beginning from day 0. The samples were shaken every 30 min between 7.00 and 19.00 h daily. At the end of each period of treatment, 60 mL of the medium was decanted into a measuring cylinder and the in-situ and other chemical parameters were determined.

### 2.6. Laboratory Analysis

#### 2.6.1. In-Situ Analysis

All the in-situ parameters were carried out following the manufacturers’ guides. The pH was determined in-situ by using a pH meter (HI9813, Hanna Instruments, Inc., Bucharest, Romania) following 3-point calibration (7.00 pH, 4.00 pH, and 10.01 pH). The turbidity and dissolved oxygen were measured using a turbidity meter (HI93414, Hanna Instruments, Inc., Bucharest, Romania) and a dissolved oxygen meter (Model: 407510, EXTECH, Taipei, Taiwan). Total dissolved solid, conductivity, salinity, and temperature were determined using a multipurpose meter (EC400, EXTECH, Taipei, Taiwan).

##### Determination of pH

The pH was determined in-situ by using a pH meter. The pH electrode was first calibrated at pH 4, 7, and 10 with pH buffers and stabilized in diluted water. The calibrated electrode was then dipped in water samples. The pH readings were taken when a stable reading was obtained.

##### Determination of Turbidity

Turbidity was measured using a digital spectrophotometric meter. The sample was poured into the turbidity meter to the desired level indicated on the bottle. The bottle was cleaned with oily soft tissue which was then inserted into the meter. The meter was turned on and calibrated, and the reading was taken. The value was expressed as nephelometric turbidity units (NTU).

##### Determination of Electrical Conductivity, Salinity, Temperature, and Total Dissolved Solid

A digital multiparameter meter was used to measure electrical conductivity, total dissolved solids, and salinity. The multiparameter was first calibrated with the appropriate buffers. The meter was dipped into the sample and the mode key in the meter was pressed and held until the unit of each parameter appeared. The parameter was recorded based on the units, which are mS/cm, g/L and, ppt for electrical conductivity, total dissolved solids, and salinity, respectively. At each mode, the temperature of the water that displayed at the base of the equipment was recorded accordingly in degrees Celsius.

##### Determination of Dissolved Oxygen

The dissolved oxygen meter was calibrated following the manufacturer’s instructions, ensuring that the sensor of the meter was disconnected. Then, the meter was turned on and the O_2_/DO selector was slid to the O_2_ position. The zero key was pressed to null the meter. The DO sensor was connected to the top of the meter (the plastic probe head protective cap was removed). The meter was allowed to stand for about 5 min until the display stabilized. The O_2_ Cal key was pressed and the display indicated approximately 20.9 (typically O_2_ in air). Then, the reading was taken by immersing the probe in the solution, and it was agitated. The reading was taken when the display stabilized.

#### 2.6.2. Determination of Nutrient (Phosphate, Sulphate, and Nitrate) and Chemical Oxygen Demand

Nitrate, phosphate, and sulphate were determined using the ultraviolet spectrophotometric screening method (APHA 4500-NO_3_-B), ascorbic acid method (APHA 4500-PE), and turbidimetric method (APHA 4500-SO_4_^2-^E). Chemical oxygen demand was determined through the open reflux method (APHA 5220-B). This analysis was carried out following the methodology previously described by APHA [38].

### 2.7. Statistical Analysis

IBM SPSS software version 20 was used to carry out the statistical analysis. Data was expressed as mean ± standard deviation. One-way analysis of variance was carried out at *p* = 0.05, and Waller–Duncan statistics was used to discern the source of the observed differences. The Spearman rho correlation matrix was used to identify the relationship between the physicochemical parameters under study.

## 3. Results and Discussion

**Figure 1:**
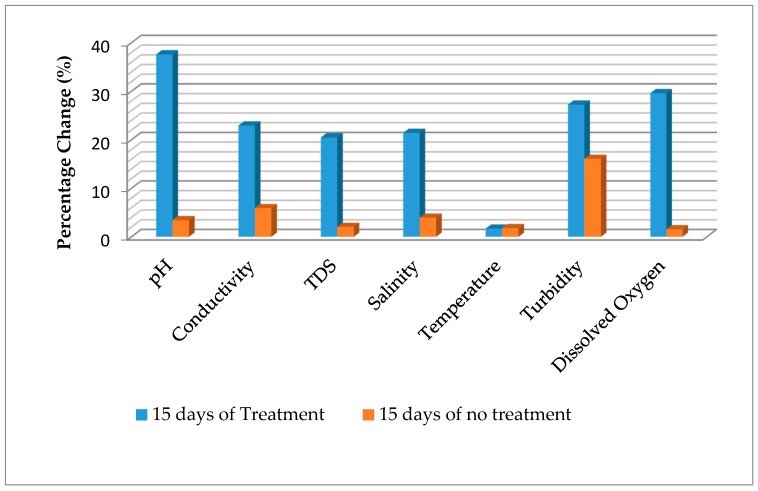
Percentage change in in-situ parameters of cassava mill effluents after 15 days.

The in-situ characteristics of cassava mill effluents from a smallholder cassava processing mill in a rural community in Delta state, treated with *S. cerevisiae* within a period of 15 days are presented in Table 1. The correlations matrix of in-situ, chemical oxygen demand (COD), and anionic characteristics of the treated effluents is presented in Table 2. The percentage changes in the in-situ parameters are presented in Figure 1.

### 3.1. pH

The pH of the water before treatment (3.93) was below the discharge limit of effluents to be discharged into surface water and land as specified by [8]. As the treatment proceeds, the pH tends towards alkalinity (Figure 1). After 15 days of treatment, the pH was 6.30. Typically, there was significant variation (*p* < 0.05) among the various days of treatment. Statistically, there was no significant difference (*p* > 0.05) between the effluent at day 0 and control after 15 days without treatment. The pH showed negative significant relationships with conductivity (r = 0.850, *p* < 0.01), total dissolved solid (r = 0.849, *p* < 0.01), and salinity (r = 0.944, *p* < 0.01), and positively correlated with turbidity (r = 0.878, *p* < 0.01) and dissolved oxygen (r = 0.947, *p* < 0.01) (Table 2). The initial effluent value (3.93) is comparable to the value of 2.50–4.20 [7], 4.1 [6], 3.96 [3], and less than the value of 5.07 reported by Orhue et al. [4]. After 15 days of treatment, the effluent was within the value of 6–9, recommended for effluent to be discharged as specified by [8]. The trend reported in this study is comparable to the work of previous authors. For instance, Iwuagwu and Ugwanyi [34] reported an increased pH in palm oil mill effluents treated with *Saccharomyces*, *Pichia*, and *Candida* species from 3.9 to near 8, depending on microbial species within a period of 0 to 168 h. Changes in pH in treated and untreated effluents after 15 days were 37.62% and 3.44% respectively (Figure 1). This variation demonstrated the efficacy of *S. cerevisiae* in the treatment of cassava mill effluents prior to discharge.

### 3.2. Electrical Conductivity

Electrical conductivity had an initial value of 14.37 mS/cm prior to treatment, which declined to 11.07 mS/cm when treated with *S. cerevisiae*, and 13.52 mS/cm without treatment (control) after 15 days. Typically, as the treatment proceeds, the conductivity declined (Figure 1). There was significant variation (*p* < 0.05) among the various days of treatment. Statistically, there was a significant decline in the control compared to the initial (Day 0) value. Conductivity showed a negative significant relationship with turbidity (r = 0.861, *p* < 0.01) and dissolved oxygen (r = 0.886, *p* < 0.01), and positively correlates with total dissolved solid (r = 0.850, *p* < 0.01) and salinity (r = 0.999, *p* < 0.01) (Table 2). The variation among the different periods of treatment could be due to changes in the holding time of the parameters. As such, it is often recommended that the conductivity of a sample be analyzed in-situ. The conductivity value reported in this present study is higher than the value of 1550 µS/cm reported by Patrick et al. [6]. The trend reported in this study is contrary with the work of Okoduwa et al. [29], which reported that 100% tannery effluents treated with *S. cerevisiae* had conductivity increased from 412 µS/cm at 0 days to 527 µS/cm after 14 days, and finally decreased to 248 µS/cm after 21 days. The authors also reported that 100% tannery effluents treated with *T. delbrueckii* increased from 412 µS/cm at 0 days to 585 µS/cm days after 14 days, and finally decreased to 476 µS/cm after 21 days of treatment. Furthermore, the authors reported dissimilar trends when different concentrations of the tannery effluent were treated by a syndicate organism (of *S. cerevisiae* and *T. delbrueckii*), and results showed a decline from 665 µS/cm at 0 days to 412 µS/cm after 21 days (100% effluent), 633 µS/cm at 0 days to 378 µS/cm after 21 days (75% effluents), and 643 µS/cm at 0 days to 399 µS/cm after 21 days. Also, Abioye et al. [35] reported an increasing trend in conductivity during the treatment of Pharmaceutical effluent by *S. cerevisiae* and *T. delbrueckii*. However, this was comparable to the work of Abioye et al. [36], which reported a decline in conductivity of textile effluents treated with *C. zeylanoides* and *S. cerevisiae* after 15 days. The decline in conductivity observed in this study could be due to reduction in nutrients, especially cations. Changes in the electrical conductivity in treated and untreated effluents after 15 days were 22.96% and 5.92%, respectively. The reduction in conductivity is similar to the findings of Okoduwa et al. [29] on tannery effluent using *T. delbrueckii*, but lower than the results using *S. cerevisiae* and the syndicate organism of *S. cerevisiae* and *T. delbrueckii*. The difference showed the effect of *S. cerevisiae* in the treatment of cassava mill effluents prior to discharge.

### 3.3. Salinity

The initial value of salinity in the effluents was 7.09 ppt, which decreased to 5.57 ppt when treated with *S. cerevisiae*, and 6.81 ppt without treatment (control) after 15 days. Like conductivity, as the treatment proceeds, the salinity concentration declined (Figure 1). There was a significant difference (*p* < 0.05) among the various days of treatment. Statistically, there was a significant decline in the control compared to the initial (Day 0) value. Salinity showed negative significant relationships with turbidity (r = 0.851, *p* < 0.01) and dissolved oxygen (r = 0.881, *p* < 0.01) (Table 2). This could be due to changes in the holding time of the parameters. As such, salinity of water and effluents is often analyzed in-situ. Salinity is relatively the amount of salt concentration in the waste water. The decrease in concentration observed in this study could be due to decline in nutrients, especially cations. Changes in salinity in treated and untreated effluents after 15 days were 21.44% and 3.95%, respectively (Figure 1).

### 3.4. Total Dissolved Solid

Total dissolved solid had an initial value of 9.76 g/L prior to treatment, which reduced to 7.76 g/L when treated with *S. cerevisiae* and 9.56 g/L without treatment (control) after 15 days. As the treatment period increased, the total dissolved solid declined (Figure 1). Significant variation (*p* < 0.05) among the various days of treatment apart from day 0, day 5, and control after 15 days showed no significance difference (*p* > 0.05), though there was an apparent decline at day 5 and control after 15 days. Total dissolved solid showed a negative significant relationship with turbidity (r = 0.807, *p* < 0.01) and dissolved oxygen (r = 0.767, *p* < 0.01), and positively correlated with salinity (r = 0.849, *p* < 0.01) (Table 2). The initial total dissolved solid in the effluents was higher than the permissible concentration of 2000 mg/L in effluents to be discharged into land and soil, as recommended by [8]. The total dissolved solid value reported in this present study is higher than the value of 799 mg/L reported by Orhue et al. [4]. The trend reported in this study is comparable with the work of Okoduwa et al. [29], which reported a decline in total dissolved solid from 248 mg/L to 130 mg/L after 21 days (100% tannery effluents), 265 mg/L to 128 mg/L after 21 days (75% tannery effluents), and 253 mg/L to 123 mg/L after 21 days (50% tannery effluents) when treated with *S. cerevisiae*; 248 mg/L to 128.3 mg/L after 21 days (100% tannery effluents), 265 mg/L to 137.5 mg/L after 21 days (75% tannery effluents), and 253 mg/L to 131.6 mg/L after 21 days (50% tannery effluents) when treated with *T. delbrueckii.* Furthermore, Abioye et al. [36] reported a decline in total dissolved solid during the treatment of textile effluents from 7.3 g/L to 5.57 g/L using *C. zeylanoides*, 6.42 g/L to 5.17 g/L using *S. cerevisiae*, and 6.41 mg/L to 4.57 g/L using a consortium of *C. zeylanoides* and *S. cerevisiae* after 15 days. The results of this study are contrary to the work of Abioye et al. [35], which reported an increase in total dissolved solid of pharmaceutical effluents from 254 mg/L to 349 mg/L after 10 days and finally decreased to 320 mg/L after 15 days when treated with *S. cerevisiae*, 254 mg/L to 382 mg/L after 10 days and finally decreased to 319 mg/L after 15 days when treated with *T. delbrueckii*, and 254 mg/L to 444 mg/L after 10 days and finally decreased to 333 mg/L after 15 days when treated with the consortium (of *T. delbrueckii* and *S. cerevisiae*). The variation suggests that the physicochemical constituents of the effluents and choice of microbes used in treatment could determine the degradation potentials. The decrease in total dissolved solid observed in this study could be due to reduction in nutrients due to degradation. Total dissolved solid in treated and untreated effluents after 15 days were 20.49% and 2.05%, respectively (Figure 1). The reduction in total dissolved solid is contrary to the findings of Okoduwa et al. [29] on the treatment of tannery effluent using *T. delbrueckii*, *S. cerevisiae*, and syndicate organism of *S. cerevisiae* and *T. delbrueckii*.

### 3.5. Dissolved Oxygen

The dissolved oxygen was 2.70 mg/L, 2.07 mg/L, 1.93 mg/L, 1.93 mg/L, and 2.40 mg/L at the initial (day 0), after 5 days, 10 days, 15 days of treatment, and 15 days without treatment (control), respectively. Typically, there was no significant difference (*p* > 0.05) among the various days except for 0 days and 15 days of no treatment (control), which was the source of the significant difference (*p* < 0.05) observed. As the treatment period increased, the dissolved oxygen content decreased (Figure 1). The results of this study with regard to initial dissolved oxygen content are similar to the work of Rim-Rukeh [7], which reported a dissolved oxygen content of 1.10–2.60 mg/L in cassava mill effluents stored for 0–50 days. The decrease in dissolved oxygen suggests that the oxygen content is being utilized by the *S. cerevisiae* as treatment progressed. Change in dissolved oxygen was 29.63% and 1.55% for treated and untreated effluents, respectively, after 15 days (Figure 1).

### 3.6. Turbidity

The initial turbidity value of the cassava mill effluents was 854.33 NTU, which increased to 1020.67 NTU, 1095.67 NTU, and 1174.67 NTU after treatment at 5 days, 10 days and 15 days, respectively. There was significant difference (*p* < 0.05) among the various treatment days, except for 10 and 15 days of treatment, which showed no significant variation (*p* > 0.05). The control showed a significant decline from the initial value. Turbidity showed positive significant relationship with dissolved oxygen (r = 0.877, *p* < 0.01) (Table 2). The turbidity trend reported in this study is contrary to the work of Okoduwa et al. [29], which reported a decline in turbidity levels of tannery effluents treated with *S. cerevisiae*, *T. delbrueckii*, and syndicate of *S. cerevisiae* and *T. delbrueckii.* Furthermore, Abioye et al. [35] reported a decrease in turbidity of pharmaceutical effluents treated with *S. cerevisiae*, *T. delbrueckii*, and the consortium. Abioye et al. [36] reported a decline in textile effluents treated with *C. zeylanoides* and an increase in the same effluent when treated with *S. cerevisiae* after 15 days. Again, the difference suggests the variation in physicochemical constituents of the effluents. The increase in turbidity level in this study could be due to increased proliferation of *S. cerevisiae* while utilizing the nutrient found in the cassava mill effluents. Turbidity in treated and untreated effluents after 15 days was 27.27% and 16.07%, respectively (Figure 1). The percentage change in turbidity is far lower than the values reported by Okoduwa et al. [29] on tannery effluents treated with *T. delbrueckii*, *S. cerevisiae*, and syndicate organism of *S. cerevisiae* and *T. delbrueckii*.

### 3.7. Temperature

The temperature readings were 27.67 °C, 27.57 °C, 27.60 °C, 27.20 °C, and 27.17 °C at the initial (day 0), after 5 days, 10 days, 15 days of treatment, and 15 days without treatment (control), respectively. Typically, there was no significant difference (*p* > 0.05) among the various days. This suggests that temperature did not affect the treatment processes. The initial temperature of the effluents is lower than the limit of <40 °C in effluents to be discharged onto land and surface water, as recommended by FEPA [8]. The findings of this study had a similar trend with previous work on the fermentation processes of several produce. Okowa et al. [39] reported that temperature did not affect fermentation of maize used in ogi (pap) production. Kigigha and Kombo [40] also reported that temperature did not affect the fermentation dynamics of guinea corn. Typically, temperature reflects the condition of the environment with regard to coldness and hotness. The temperature of the treatment cassava mill effluents is similar to the ambient temperature of area where the study was carried out. Slight apparent changes in temperature exist and were 1.70% and 1.81% for treated and untreated effluent, respectively, after 15 days.

**Figure 2:**
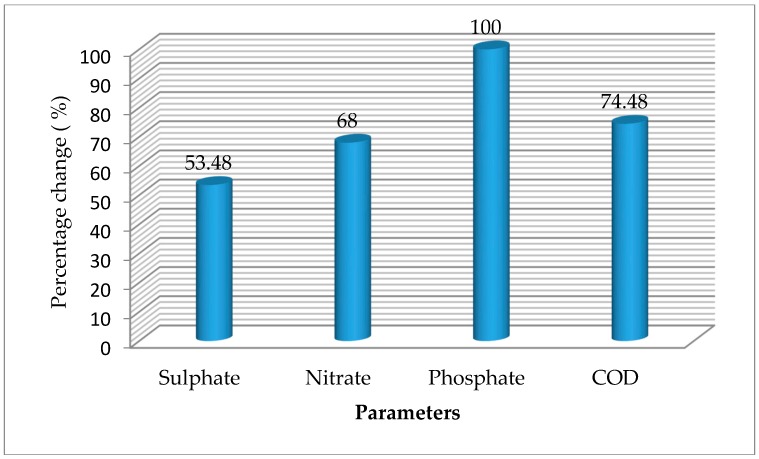
Percentage change in chemical oxygen demand (COD) and anionic parameters of cassava mill effluents after 15 days.

The chemical oxygen demand and anionic characteristics of cassava mill effluents from a smallholder cassava processing mill in a rural community in Delta state, treated with *S. cerevisiae* within a period of 15 days, are presented in Table 3, while the changes resulting from treatment of the effluent by *S. cerevisiae* are presented in Figure 2.

### 3.8. Chemical Oxygen Demand (COD)

The COD of the effluents before treatment was 1663.33 mg/L (Table 3). As the treatment proceeds, the COD declined (Figure 2). After 15 days of treatment, the COD was 425.00 mg/L. There was significant variation (*p* < 0.05) among the various days of treatment. COD showed negative significant relationship with pH (r = 0.923, *p* < 0.01), turbidity (r = 0.804, *p* < 0.01), and dissolved oxygen (r = 0.881, *p* < 0.01), and positively correlated with conductivity (r = 0.971, *p* < 0.01), total dissolved solid (r = 829, *p* < 0.01), and salinity (r = 0.978, *p* < 0.01) (Table 2). The decline in the COD suggests the effect of *S. cerevisiae* in the degradation of the cassava mill effluents. The value reported on the initial day (1663.33 mg/L) is higher than the value of 320–365 mg/L previously reported in effluents stored for 0–50 days, as reported by Rim-Rukeh [7]. The trend reported in this study is comparable to the work of previous authors. For instance, Iwuagwu and Ugwanyi [34] reported a decline in COD from 114,800 mg/L (at the initial days) to less than 25 g/L when treated with *Saccharomyces, Pichia*, and *Candida* species. Abioye et al. [36] reported a decline in COD of textile effluents treated with *C. zeylanoides* and *S. cerevisiae* after 15 days. The change in COD was 74.48% in this study, higher than the value of 52.5% reported in pharmaceutical effluents treated with *S. cerevisiae* [35] and the value of 54.2% when *S. cerevisiae* was used to treat tannery effluents [29]. The variation in percentage change could be due to constituents of the effluents, as well as the treatment period.

### 3.9. Sulphate

The sulphate level prior to treatment was 79.470 mg/L, which reduced to 36.967 mg/L after 15 days of treatment with *S. cerevisiae*. Basically, there was significant variation (*p* < 0.05) among the various days of treatment (Table 3). Furthermore, there was no significant difference (*p* > 0.05) between the effluent at day 0 and day 5. Sulphate showed positive significant relationships with phosphate (r = 0.542, *p* < 0.05) and temperature (r = 0.558, *p* < 0.05) (Table 2). The initial sulphate concentration is lower than the permissible level of effluent to be discharged into surface water (500 mg/L) and land (1000 mg/L), as recommended by FEPA [8]. The sulphate concentration reduced by 53.48% after 15 days of treatment, suggesting the efficacy of *S. cerevisiae* in the degradation of the cassava mill effluents (Figure 2). When the test is compared to the control, the fluctuation could be due to the influence of environmental factors, such as temperature. In this study, sulphate showed positive significant relationships with temperature and phosphate. The trend reported in this study is lower than the value of 83.0% tannery effluents treated with *S. cerevisiae* [29]. Abioye et al. [36] reported a decline in sulphate of textile effluents treated with *C. zeylanoides* and *S. cerevisiae* after 15 days, which has some similarity to the trend reported in this present study. The authors reported a decline in sulphate during the treatment of textile effluents from 30 mg/L to 19 mg/L using *C. zeylanoides*, 57 mg/L to 28 mg/L using *S. cerevisiae*, and 28 mg/L to 16 mg/L using a consortium of *C. zeylanoides* and *S. cerevisiae* after 15 days. The variation in percentage change could be due to the constituents of the effluents, as well as the treatment period.

### 3.10. Nitrate

The initial nitrate concentration was 250.00 mg/L, which reduced to 80.00 mg/L after 15 days of treatment with *S. cerevisiae*. Basically, there was significant variation (*p* < 0.05) among the various days of treatment apart from 0 and 5 days, which had no significant difference (*p* > 0.05) (Table 3). Nitrate showed positive significant relationships with phosphate (r = 0.542, *p* < 0.05), temperature (r = 0.822, *p* < 0.01), COD (r = 0.589, *p* < 0.05), conductivity (r = 0.596, *p* < 0.05), and salinity (r = 0.592, *p* < 0.05), and negatively correlated with dissolved oxygen (r = 0.552, *p* < 0.05) (Table 2). The initial nitrate concentration was lower than the permissible level of effluent to be discharged into surface water (20.00 mg/L), as recommended by FEPA [8]. Nitrate concentration reduced by 68.00% after 15 days of treatment, suggesting the efficacy of *S. cerevisiae* in the degradation of the cassava mill effluents (Figure 2). The trend reported in this study is lower than the value of 58.7% in pharmaceutical effluents treated with *S. cerevisiae* [35]. The trend is also comparable to the work of Abioye et al. [36], which reported a decline in textile effluents treated with *C. zeylanoides* and *S. cerevisiae* after 15 days. The variation in percentage change could be due to the constituents of the effluents, as well as the treatment period.

### 3.11. Phosphate 

The phosphate level at the beginning of the experiment (day 0) was 35.00 mg/L, which reduced to 0.00 mg/L after 15 days of treatment with *S. cerevisiae*. Basically, there was significant variation (*p* < 0.05) among the various days of treatment, apart from 5 and 15 days, which had no significant difference (*p* > 0.05) (Table 3). The initial phosphate concentration was higher than the limits of 5 mg/L and 10 mg/L for effluents discharged into surface water and land, respectively, as recommended by FEPA [8]. Phosphate concentration reduced by 100.00% after 15 days of treatment, meaning that *S. cerevisiae* has effects on cassava mill effluents (Figure 2). Like sulphate, this fluctuation/trend could be associated to environmental factors, such as temperature. The trend reported in this study is higher than the value of 60.5% in tannery effluents treated with *S. cerevisiae* [29]. Abioye et al. [35] reported a decline in phosphate concentration of pharmaceutical effluents from 3.7 mg/L to 1.6 mg/L after 10 days and finally increased to 5.82 mg/L after 15 days when treated with *S. cerevisiae*, 3.7 mg/L to 1.45 mg/L after 10 days and finally increased to 2.28 mg/L after 15 days when treated with *T. delbrueckii*, and 3.7 mg/L to 1.57 mg/L after 5 days and finally increased to 1.92 mg/L after 10 days when treated with the consortium (of *T. delbrueckii* and *S. cerevisiae*). Furthermore, Abioye et al. [36] reported a decline in phosphate content of textile effluents treated with *C. zeylanoides* and *S. cerevisiae* after 15 days.

The reduction in some parameters with treatment could be attributed to the removal of organic load and toxicity from the effluents [29,35,41], while the significant increase in some parameters may be associated to the saturation of the organisms binding site with such characteristics and death of some of the organisms [29,35]. Furthermore, Abioye et al. [35] also attributed increase in some parameters, such as nitrate, phosphate, chemical oxygen demand, and turbidity in pharmaceutical effluent treated with *S. cerevisiae*, *T. delbrueckii*, and the consortium to the presence of the yeast.

## 4. Conclusions

Nigeria is the largest cassava-producing nation. During processing, large volumes of water are generated at the dewatering zone. This wastewater is usually discharged into the environment with little or no treatment. This study investigated the effect of *S. cerevisiae* in the treatment of the physicochemical characteristics of the wastewater. The study found that *S. cerevisiae* on cassava mill effluent leads to a reduction in acidity of pH (tending toward alkalinity), conductivity, total dissolved solid, salinity, sulphate, nitrate, phosphate, dissolved oxygen, and COD, and an increase in turbidity. This showed that *S. cerevisiae* has a positive effect toward sustainable management of cassava mill effluents.

## Figures and Tables

**Table 1:** In-situ concentration of cassava mill effluents treated with *Saccharomyces cerevisiae*.

Table. *Cont.*	Initial (Day 0) Post Treatment	5 days Post Treatment	10 days Post Treatment	15 days Post Treatment	Control after 15 Days
pH	3.93 ± 0.06 a	4.93 ± 0.15 b	5.33 ± 0.06 c	6.30 ± 0.10 d	4.07 ± 0.06 a
Conductivity, mS/cm	14.37 ± 0.31 e	12.94 ± 0.12 c	12.34 ± 0.24 b	11.07 ± 0.07 a	13.52 ± 0.10 d
TDS, g/L	9.76 ± 0.12 c	9.44 ± 0.43 c	8.55 ± 0.30 b	7.76 ± 0.16 a	9.56 ± 0.10 c
Salinity, ppt	7.09 ± 0.05 e	6.52 ± 0.19 c	5.99 ± 0.05 b	5.57 ± 0.13 a	6.81 ± 0.10 d
Temperature, °C	27.67 ± 0.60 a	27.57 ± 0.38 a	27.60 ± 0.20 a	27.20 ± 0.10 a	27.17 ± 0.12 a
Turbidity, NTU	854.33 ± 58.16 b	1020.67 ± 12.34 c	1095.67 ± 8.50 d	1174.67 ± 18.04 d	717.00 ± 5.57 a
Dissolved oxygen, mg/L	2.70 ± 0.10 c	2.07 ± 0.12 a	1.93 ± 0.06 a	1.90 ± 0.10 a	2.40 ± 0.10 b

Data is expressed as mean ± standard deviation; Different letters across the row indicate significant difference (*p* < 0.05), according to Waller–Duncan statistics; TDS = total dissolved solid; nephelometric turbidity units (NTU).

**Table 2:** Spearman’s rho of in-situ, chemical oxygen demand (COD), and anion concentration of cassava mill effluents treated with *Saccharomyces cerevisiae*.

Parameters	Sulphate (mg/L)	Nitrate (mg/L)	Phosphate (mg/L)	COD (mg/L)	pH	Conductivity (mS/cm)	TDS (g/L)	Salinity	Temperature (°C)	Turbidity (NTU)	Dissolved Oxygen (mg/L)
Sulphate	1.000	-	-	-	-	-	-	-	-	-	-
Nitrate	0.452	1.000	-	-	-	-	-	-	-	-	-
Phosphate	0.542 *	0.822 **	1.000	-	-	-	-	-	-	-	-
COD	0.014	0.589 *	0.431	1.000	-	-	-	-	-	-	-
pH	0.129	−0.620 *	−0.410	−0.923 **	1.000	-	-	-	-	-	-
Conductivity	−0.100	0.596 *	0.443	0.971 **	−0.953 **	1.000	-	-	-	-	-
TDS	−0.133	0.429	0.294	0.829 **	−0.849 **	0.850 **	1.000	-	-	-	-
Salinity	−0.083	0.592 *	0.444	0.978 **	−0.944 **	0.999 **	0.849 **	1.000	-	-	-
Temperature	0.558 *	0.272	0.460	0.111	−0.026	0.063	0.341	0.076	1.000	-	-
Turbidity	0.366	−0.461	−0.097	−0.804 **	0.878 **	−0.861 **	−0.807 **	−0.851 **	0.140	1.000	-
Dissolved oxygen	0.143	−0.552 *	−0.265	−0.881 **	0.947 **	−0.886 **	−0.767 **	−0.881 **	0.031	0.877 **	1.000

* Correlation is significant at the 0.05 level (2-tailed). ** Correlation is significant at the 0.01 level (2-tailed), N = 15, n = 3.

**Table 3:** Chemical Oxygen demand (COD) and some Anion concentration of cassava mill effluents treated with *S. cerevisiae*.

Days	Sulphate, mg/L	Nitrate, mg/L	Phosphate, mg/L	COD, mg/L
0 post treatment	79.470 ± 4.056 c	250.000 ± 14.731 d	35.000 ± 6.000 c	1663.333 ± 387.043 c
5 post treatment	91.700 ± 16.899 c	105.000 ± 15.309 b	0.000 ± 0.000 a	862.533 ± 88.950 b
10 post treatment	176.300 ± 6.474 d	240.000 ± 7.000 d	20.000 ± 2.166 b	700.000 ± 31.000 ab
15 post treatment	36.967 ± 7.323 b	80.000 ± 6.900 a	0.000 ± 0.000 a	425.000 ± 46.360 a
15 no treatment (Control)	0.000 ± 0.00 a	150.000 ± 12.490 c	0.000 ± 0.000 a	900.817 ± 88.228 b

(Data is expressed as mean ± standard deviation; Different letters along the column indicate significance difference (*p* < 0.05) according to Waller–Duncan statistics).
